# Why environmentalists eat meat

**DOI:** 10.1371/journal.pone.0219607

**Published:** 2019-07-11

**Authors:** Evon Scott, Giorgos Kallis, Christos Zografos

**Affiliations:** 1 ICTA-UAB, Universitat Autonoma de Barcelona, Barcelona, Spain; 2 ICREA, Barcelona, Spain; 3 John Hopkins University – Universitat Pompeu Fabra (JHU-UPF) Public Policy Centre, Barcelona, Spain; 4 Research Group on Health Inequalities, Environment, Employment Conditions Network (GREDS-EMCONET), Department of Political and Social Sciences, Universitat Pompeu Fabra, Barcelona, Spain; Universidade do Porto Instituto de Biologia Molecular e Celular, PORTUGAL

## Abstract

Why do people who care about the environment adopt behaviours that are not consistent with their beliefs? Previous studies approach this as a case of cognitive dissonance, researchers looking into the strategies through which people reduce gaps between their attitudes and their behaviours. Here we start from the premise that there is no dissonance, and that people have consistent reasons of why they are doing what they are doing. The research task is then to shed light on these reasons. Using Q-methodology, a mixed quantitative-qualitative approach, we interviewed 42 environmentally-minded researchers asking them why they eat meat. Our interviewees were aware of and cared about the environmental and ethical impacts of meat eating, but reasoned that they eat meat because either technological, or political changes are more important than what they personally do, because of doubts about the impact of personal action in a complex world, or simply because they lack the determination to stop eating meat. Our analysis suggests that policies and messages that try to educate or guilt meat-eaters are unlikely to work with those well aware of the impacts of their actions.

## Introduction

People often say one thing about the environment and do another. The ‘meat paradox’ refers to the observation that many people who care for animal welfare and are concerned with the impacts of meat production, follow meat diets with harmful effects[1]. Such ‘attitude-behaviour’ (or belief-action) gaps are not just a matter of misinformation [2–4]. Economists for example have argued that personal preferences are revealed in consumer choices—what matters is what people do, not what they say or believe[5]. Others show how behaviours are bounded by social norms and cultural contexts, not simply determined by beliefs [6,7]. In social practice theory, actions and beliefs form together through practice [8,9]. For example, socialization and everyday practice make people reluctant to stop meat [10]. Separate research looks at individual and structural—social and institutional—constraints to behavioral change [6].

A different strand of literature is looking at how people deal with ‘cognitive dissonance’ between their beliefs and actions [11]. Research has shown that when people do not harmonize dissonance by aligning behavior to beliefs (e.g. stop eating meat if they are environmentalists), they change beliefs to justify actions (e.g. deny the impacts of meat-eating) [12]. People may ‘neutralize’ unethical behaviors by denying personal responsibility or injury (e.g. deny the effects of eating meat), by condemning those who condemn them (e.g. blame vegetarians as hypocrites), or by justifying their actions in terms of a broader good (e.g. argue that meat-eating is good for the economy) [13]. We know from previous studies that meat eaters may dissociate from meat production and its environmental or animal welfare effects [14]; or justify eating meat as ‘natural, necessary, normal, or nice’ [15].

Many empirical studies in this vein try to measure the distance between environmental beliefs and actions [16], and explain the attributes that make certain people more prone to certain behaviors [17], or else identify the strategies that people use to reduce the dissonance and justify their actions. The assumption is that there is a ‘proper’ environmental behaviour, and that if someone cares for the environment and does not follow this practice, then there is dissonance. Here instead, we take a different path. We do not assume distance or dissonance, but rather that people have consistent reasons for doing what they do. We want to hear from people themselves, in this case informed people who care about the environment, how they explain their actions. Q-methodology [18], a quantitative-qualitative approach has been used by others for deciphering people’s diverse viewpoints (‘discourses´) on an issue and is ideal for elucidating how people reason about what they do, without prior assumption of consistency or dissonance. Unlike regression analysis that looks for correlations between variables across a sample of subjects, Q looks for correlations between subjects and their viewpoints across a sample of variables [19]. Strongly correlated viewpoints form an identifiable ‘discourse’–a shared way of viewing, and talking about an issue.

## Materials and methods

For our research we followed the standardised steps of Q-methodology. Q has a different logic than conventional, statistics-oriented survey research and we explain this here. These differences need to be kept in mind when evaluating our sample selection or interpreting our results (for more details of study design, analysis, and results see also Supplementary Information).

We started with a review of academic literature, media and popular texts, as well as four long interviews with experts through which we produced what in Q terminology is called the ‘concourse’–a list of 116 statements that people use to justify why they eat meat. A final sample of 30 statements was produced through formal procedures, using a two-axed ‘concourse matrix’ to sort statements, with categories based on rationality types, existing studies of meat-eating, and by open coding extraneous statements. (see full list in Supplementary Information). We then conducted pilot interviews and corrected some statements for clarity.

Representativeness in Q refers to representativeness of the statements (Q-set), not the type and number of individuals interviewed. Unlike standard survey and regression analysis, Q methodology establishes patterns within and across individuals and their views, not across individual traits [20]. In Q “it is not the ‘constructors’–the participants—who are the focus but the ‘constructions’ themselves” [21]. For those familiar with regression analysis, it may be helpful to think of interviewees as the variables and Q statements as the cases. The method does not require then large numbers of participants to produce valid results [22]. The objective is not to interview a sample representative of a larger population, but a focused, yet varied set of people with well-informed but different views to capture the diversity in the universe of thoughts as much as possible.

We interviewed forty-two meat-eaters from our own research institute (ICTA-UAB), the reasoning being that environmental researchers care about the environment and know the impacts of meat-eating and so fit to the study’s purpose of inquiring into the meat-paradox. Unlike previous studies that have focussed on the meat-eating motivations of the population at large, we were interested to see here how people that one would think more prone to being vegetarian, justify their choice to eat meat. Researchers at ICTA-UAB fit this profile perfectly, with the added advantage that they have a diversity of educational backgrounds, fields, nationalities, ages and politics, which is an important property for a Q-study that seeks to capture the variety of discourses at stake (see Table 1). We ensured that this diversity was reflected in the sample of people that we interviewed.

**Table 1:** Characteristics of our sample.

	Number	%
**Professional Status**		
Interns	2	4.8
Masters Students	5	11.9
PhD Candidates	16	38.1
Post-Doctoral Candidates	9	21.4
Professors	10	23.8
**Times meat consumed/week**	3.5	50.0
**Age Group**		
20–29	19	45.2
30–39	12	28.6
40–49	3	7.1
50–59	4	9.5
60–65	4	9.5
**Average Age**	36	
**Gender**		
Female	18	43
Male	24	57
**Country of Origin**		
Spain	24	57.1%
U.S.	4	9.5%
Brazil	2	4.8%
Canada	2	4.8%
Italy	2	4.8%
Mexico	2	4.8%
France	1	2.4%
Germany	1	2.4%
Greece	1	2.4%
India	1	2.4%
Italy/Scotland	1	2.4%
Peru	1	2.4%

It is worth noting that a Q study should not be interpreted as a survey—for which obviously a sample of forty-two researchers from our own institute would be biased and of limited significance. Instead, a Q-study elucidates different discourses at stake—and for discerning such differences researchers in our institute are a good basis (bearing of course the limitations that we discuss at the end of the paper, in extrapolating differences observed in our sample to all environmentally-aware meat eaters).

We interviewed each researcher separately and gave her or him 30 cards, each with one statement, asking to value them from most unlike (-4) to most like (+4) their view in a Q grid of a forced, normal distribution shape. Each participant produced hence what is called in the literature a ‘Q sort’–a ranking of statements. Interviewees could rearrange the resulting rank in an order that best expressed their opinion (Q-methodology allows for this, because it recognizes that people often think about ideas in relation to one another and not in isolation). Participants were then interviewed, asked about highest, lowest, or unusual choices (meaning those very different when compared to previously observed sorts) and welcomed to offer more comments about their choices. The interviews were recorded and transcribed, and salient quotes were used to support the factor analysis.

Q-methodology generates distinct discourses through factor analysis. Data analysis was carried out using PQ-Method, a software for Q-studies [23]. As most Q-studies, we used Principal Component Analysis (PCA) and Varimax rotation [19]. PCA extracts distinct factors (representing discourses) based on a correlation matrix between Q-sorts. Each Q-sort’s level of correlation with each factor is then calculated, indicating the respondent’s level of agreement with that viewpoint. The factors are ‘rotated’ to clarify viewpoints [24]. Varimax statistical rotation maximizes the variance across factors. Sorts are then ‘flagged’—it is decided whether they will be included in the final computation of factors. Sorts which correlate too highly with multiple factors, or with no factors, are excluded. Flagging was first done automatically but then manually inspected and corrected, applying a threshold based on a standard formula (2.58(1/√n), where n = the total number of statements) and recommended procedures in Q. Finally, the representative sort for each discourse is computed (with values of +1, +2 etc.) along with statistically significant distinguishing and consensus statements, providing the basis for interpreting the discourses.

The above process was carried out in examining solutions of 2–7 factors. Ultimately we decided on a 4-factor solution (see Table 2 with 4 discourses). Other solutions produced factors with no significantly correlating sorts, sorts which correlated significantly with multiple factors, sorts which correlated with no factors, or factors which did not appear meaningful on closer inspection. Another major concern was that other solutions produced higher interfactor correlations, meaning less distinctive viewpoints.

**Table 2:** Four discourses and their distinguishing statements.

No.	Distinguishing Statements per Factor	Score	Z-score
***Discourse 1 (D1)*. *Optimism*: *“The future will solve it*.*”***			
22*	I realize I have to stop in the future.	+3	1.533
19*	Technology will solve the environmental and animal welfare problems of meat production (ex: in vitro meat).	+3	1.414
30**	Meat is delicious.	+1	0.464
9*	Other animals eat each other and it’s natural. Humans are also part of the life-cycle.	0	-0.209
15**	We have evolved to eat meat and have the biology of omnivores	-1	-0.501
***Discourse 2 (D2) System-Focus*: *“It’s the system*, *not the meat*.*”***			
5*	The environmental impacts of eating meat are exaggerated.	+3	1.500
16**	Enjoying meat with others is important to me.	+3	1.381
18**	Changing industrial farming is a matter of political change, not individual choice.	+2	1.126
15**	We have evolved to eat meat and have the biology of omnivores.	0	0.235
1*	Animals don't suffer as much as we do because they are less complex organisms	0	-0.030
29**	Eating meat shows that we are affluent in our society.	-3	-1.723
19**	Technology will solve the environmental and animal welfare problems of meat production (ex: in vitro meat).	-4	-1.791
***Discourse 3 (D3)*. *Complexity*: *“It is my moral responsibility*, *but it’s more complex than that…”***			
4*	A lot of people would lose their jobs if we all stopped eating meat.	+1	0.398
2**	It would be too expensive for me to eat well and be vegetarian	0	-0.000
19*	Technology will solve the environmental and animal welfare problems of meat production (ex: in vitro meat).	0	-0.382
18**	Changing industrial farming of meat is a matter of political change, not individual choice.	-1	-0.453
5*	The environmental impacts of eating meat are exaggerated.	-1	-0.616
20*	It would be socially awkward for me to stop eating meat.	-3	-1.408
1*	Animals don't suffer as much as we do because they are less complex organisms	-4	-2.365
***Discourse 4 (D4)*. *Feebleness*: *“I should*, *but I lack the willpower*.*”***			
30*	Meat is delicious.	+4	2.025
24**	Because I’m distant from food animals it´s hard to connect my consumption and their suffering.	+2	1.146
14**	There are sustainable alternatives to industrial farming.	+1	0.846
8**	Widespread vegetarianism would have a negative impact on the environment. (For example, hunters support conservation far more than animal rights activists.)	-2	-0.889
19**	Technology will solve the environmental and animal welfare problems of meat production (ex: in vitro meat).	-2	-1.071
23*	I don’t want to be identified as a vegan/vegetarian because they’re not likeable.	-4	-1.856

A discourse refers to a set of statements a group agreed or disagreed with in ways that are statistically significant in terms of their difference from statements in other groups (*—significance at p < .01, ** significance at p < .05). Scores indicate the average degree of agreement with a statement among interviewees who belong to this group, from– 4 (least agree with statement) to +4 (most agree). “0” means the respondent neither agrees nor disagrees.

## Results and discussion

The statistical analysis of rankings identified four discernible viewpoints or discourses, summarised in Table 2 and explained below. In line with standard Q practice, we report on statements for which there is statistically significant difference between interviewees that chose them and interviewees that did not. It is around such differences that we discern four distinguishable groups. In other words, certain statements ranked by our respondents will not show up in Table 2 as they were not statistically different among groups.

Different to Q study convention, we present a consensus statement first, because it helps see the common ground with which all four discourses develop. The consensus statement (#S7) indicates that none of the discourses differs in terms of the importance participants give to meat-eating compared to other environmental concerns: they all give it more or less the same importance—not more, nor less.

In Discourse 1 (D1), *Optimism*, respondents explained that they keep eating meat not because it is natural (#S15, #S9) or only because they like it (#S30), but mostly because change will be easier in the future (#S22, #S19)–be it because it will be easier for them to change (#S22), or because technology will overcome the negative effects of meat production (#S19). People in this group talked more than others about practical ‘solutions’–technologies and economic policies that reduce meat consumption or impacts (#I22, #I32). We characterise this discourse as an optimistic discourse that expects things to become better in the future, broadly in line with eco-modernist or technologically optimistic discourses that expect that environmental problems, however hard, can and will be solved in the future.

For the *System-Focus* discourse (D2), views seem to coalesce around the idea that the problem is the system not the meat. This discourse does not share the technological optimism of the Optimism discourse (#S19) and sees change as a matter of political change, not personal habits (#S18). Interviewees talked of ‘big businesses’ and ‘politicians or powerful people in the agro-industrial sector’ that push profit-driven technologies and make it impossible for small-scale farms to subsist (#I28, #I26). As one interviewee put it, this is ‘a political issue and an issue of capitalism… It’s worse [to not eat meat] because you convince yourself that you’re doing something good’ (#I28). For System-Focus discourse holders, eating meat is not a matter of taste or expressing wealth (#S29), but of enjoying it with others (#S16). The discourse does not reject that animals suffer less than humans (#S1), and is the only discourse that held that the impacts of meat are exaggerated (#S5), at least compared to other environmental problems or problems with other staples (one interviewee mentioned ‘child labor exploitation in soy production’ (#I31)). Respondents here reason that they eat meat, because changing their own behaviour will not make a difference, unless the system changes as a whole.

In contrast, for those sharing the *Complexity* discourse (D3) it is individual, rather than political change that matters (#S18) (also unlike System-Focus, Complexity holds that the impacts of meat-eating are not exaggerated (#S5)). This line of reasoning does not reject that price is an issue for adopting a vegetarian diet (#S2), finds little importance in engaging with the question of the capacity of technology to resolve animal welfare problems (#S19), is sensitive to the suffering of animals (#S1), rejects social pressure (#S20), and is concerned with the effects of personal action (#S4). An underlying theme for this discourse is personal responsibility. ‘Consumers should take responsibility for their choices’, one interviewee said (#I37); and another: ‘every choice has an impact, everything has an effect’ (#I11).

From the ranking and correlation of statements (Table 2), it is not clear why those who shared this discourse keep eating meat. In interviews though many attributed their inaction to ‘complexity’. This fits the agnostic position of the group regarding technology (#S19). As one respondent for example said ‘being responsible …is more complex …It’s not a yes or no. It’s how much, when, for what purpose’ (#I37). Or as another put it: ‘it’s very difficult to establish a relationship of causality between what you do and what happens. There are too many variables … It’s nice to imagine that we are being nice and saving the world but it’s complicated, especially with food” (#I2).

Unlike the first three discourses, those in the fourth believed that personal action can make a difference, albeit is hard to come by. This *Feebleness* discourse (D4) holds that although giving up eating meat makes sense, the necessary will to actually do it is not there. Feebleness accepts that there are sustainable alternatives to industrial farming (#S14), and that technology won’t solve environmental and animal suffering from industrial meat production (#S19). What is more, there is a clearly positive view of vegetarianism evident in rejecting alleged environmental impacts of widespread vegetarianism (#S8) and that a vegetarian identity can be a social drawback (#S23).

Still, distance from food animals makes it hard to connect meat consumption with their suffering (#S24). Simply, meat is delicious (#S30). Respondents realized their ‘weakness’ and admired those who give up meat. ‘Putting your ideals [above] your own pleasure…making your life difficult for your ideals is something that I admire’, affirmed one interviewee (#I25). ‘They’ve made a less selfish decision than I have’, said another (#I36).

If Optimism, System-Focus, and Complexity discourses justify inaction on the basis of the belief that it is a matter of technology, politics, or simply too complex to do anything about it, Feebleness discourse-holders accepted that they should stop eating meat, admired those that do, but conceded that they simply cannot do it. In other words, this group lives with its contradictions.

Our findings are enlightening given existing empirical studies on meat-eating. In Sedova et al., the most similar study to ours, promises of future behaviour change were more common among the environmental studies students they interviewed than in the general population [4]. This finding is echoed in the Optimism discourse of our study, supporting the idea that promises of future behaviour change are common among environmentally educated individuals.

The most widely cited articles on the ‘meat-paradox’ focus on consumer prejudice towards animals, specifically how many people deny that the animals can suffer or question their moral status [1,25]. In our environmentally-minded sample instead, such arguments (#S1, #S13) were among the ones our interlocutors most disagreed with. Again, this may be the result of the environmental profile of our participants and their education levels.

Building on previous studies typifying justifications, Piazza et al. found that the vast majority (83–91%) of justifications fell under the ‘4Ns’–that eating meat is *natural*, *necessary*, *normal*, or *nice* (i.e.: enjoyable) [15]. However, discourses 1–3 in our study emphasize systemic reasons, which differ greatly from common justifications that arose in Piazza et al.’s study and are not easily captured by such categories. Such differences support the idea that justification of meat-eating is more complex among more environmentally-aware individuals. Such samples warrant further study, especially given the increasing emphasis on environmental issues in the popular media.

## Conclusions

Previous research has assumed that if people think that eating meat is bad and they keep eating, then there must be some cognitive dissonance—studies in this line focus then on understanding the scale and drivers of this dissonance or how people alleviate it. Our approach acknowledges that subjects can have coherent reasons for their choice to eat meat. The question then is how do people reason and explain their apparently unsustainable actions given their environmental beliefs.

Rather than ignoring and denying the problem or excusing their actions, the people we interviewed here in effect question whether personal action makes a difference (Discourses 1–3). If one approaches this from a cognitive dissonance perspective, the conclusion could be that people appeal to systemic forces (technology, politics or complexity) or the future to excuse their inaction and be at peace with themselves. Our approach instead opens the possibility that subjects may well have *internalized* views that are quite common in sustainability science. For example, they may consider that barriers to sustainable agriculture are structural, causation is complex, and solutions are political or technological, rather than a matter of individual consumption.

These are all valid claims and there is no reason why they should only be seen as excuses. People may not be excusing themselves or reducing their dissonance, but truly acting in accordance with their beliefs, beliefs aligned with both caring for the effects of meat-eating and continuing to eat meat. If people believe that changing their behavior will not make much of a difference, because the solution is primarily technological or political, or a complex combination of all, then there is no paradox or dissonance to begin with. People justify coherently their choice (Discourses 1–3), or simply live with their contradictions, aware and easy with the fact that they are contradictions, unlike cases of dissonance (Discourse 4).

Q-methodology is good for identifying the variety of subjective viewpoints on an issue, but to assess how common the four views identified here are and who holds them, representative surveys and regressions are necessary. If the views identified here are prevalent, at least among environmentally minded people, then policies or messages seeking to inform these people about the environmental consequences of their actions, or to guilt them into stopping their environmentally damaging behaviour, in this case eating meat, are likely to be less effective. People in discourses 1–3 do not agree that becoming vegetarians will make a difference, while those in discourse 4 have surrendered to the fact that they cannot become vegetarians. Nudging people into certain environmental behaviours might be more difficult than previously thought, not because people don't care or because they haven’t heard about these problems and appropriate behaviours, but because they do not believe that it is a change in behaviours that will really make a difference.

## Supporting information

S1 File Supplementary information on methods and data. (DOCX)
