# Supplementary material for: Why environmentalists eat meat
Source: PLoS One. 2019 Jul 11;14(7):e0219607. doi: 10.1371/journal.pone.0219607 (PMC6622546; doi:10.1371/journal.pone.0219607)
Supplement: S1 File — (DOCX) [file pone.0219607.s001.docx]

# **Supplementary Information on Method and Data**

Given differences between Q methodology vis-à-vis other subjective viewpoint elicitation techniques and methods, we use this section to briefly introduce the methodology and in particular its approach to subjectivity. We then move on to the main focus of this section which is to explain into detail the stages and processes conducted for our study’s design, data collection, analysis and interpretation.

1. **Q methodology and the study of subjective viewpoints**

Q-methodology conducts a scientific study of subjectivity. Q uses factor analysis statistical techniques to explore subjectivity within a group, and has been used in diverse fields for over 80 years^1–4^. Q was first introduced by William Stephenson, a psychologist and physicist, in a letter to *Nature* in 1935 in response to discussions in the journal regarding techniques of factor analysis. Though it has primarily been used in psychology, in recent years, Q-methodology has seen increased application to environmental issues^5–10^.

Q is distinct from most methods using factor analysis and from Likert-scale surveys in that rather than finding correlations between variables across subjects, it finds correlations between a group of subjects across a sample of variables. It is a mixed-method approach, combining quantitative and qualitative elements, specifically designed to seek out the differences between viewpoints within a particular group. Analysis of the sample of variables is always based on a single, commensurable unit of measurement – ‘self-significance,’ whose values are assigned by the subjects themselves when they arrange the statements^2^. Q studies do not claim to produce generalizable results outside the group that studied in the same manner as R; it purports only to describe the discourses at work within a particular group, who are sampled purposively^3^. What is meant to be representative in Q is the pool of statements and not the participants selected. To question how a Q-study samples its participants is simply to question the importance of studying a particular group. If there are good reasons to study that group, then one ought not reject a Q study on such grounds.

Q is premised on a particular understanding of and approach to the study of subjectivity, namely one based on ‘operant subjectivity.’ According to proponents of Q-methodology, survey techniques, Likert scales, and in general devices that measure opinions, remove the subject’s frame of reference from inquiry. Those methods appeal to an external frame of reference (in the case of our study, models of behaviour such as the Theory of Planned Behaviour (TPB), the Value Belief Norm theory (VBN), and the Norm Activation Theory (NAM), neutralisation theory, etc.) to which the researcher appeals in order to understand or explain the subjective viewpoints of respondents, and which the researcher uses in order to measure against the respondent’s reactions to a fixed set of questions^11^. Operant subjectivity considers that subjects may be constituted in ways that a priori categories introduced by models of behaviour may fail to grasp, and explores subjectivity from respondents’ own self-referential standpoints, in the form of points of view about concrete statements^12^. Q-sorts enable this by allowing subjects to create their own picture of their point of view by responding to statements selected to represent the variability of available viewpoints about an issue, without that picture necessary corresponding to categories of reference (e.g. models of behaviour) preselected by the researcher^13^.

The main outcome of Q studies is a series of ‘discourses’ about a topic. Discourses represent sets of distinct, shared ways of understanding reality, or else different ‘worldviews’, i.e. ways of seeing, thinking and feeling about something^9^. Discourses are shared in the sense that each discourse is constructed through the contribution of several individual viewpoints that coalesce with reference to an issue; and they are distinct in the sense that each discourse is different to the rest with respect the specific viewpoints they hold towards that same issue. Examples pertaining to environmental and sustainability issues include conflictive discourses about grizzly bear conservation in Canada^14^; discourses of Global South elites about environmental and sustainability issues^15^; competing visions between ecologists about what research should be done and why^16^; and, diverse discourses held by conservationists about ‘the market’^17^.

The main stages of conducting Q-studies^8,24^ are summarised below; the details of how we conducted those stages in our study are explained in subsequent sections:

1. Study Design
   1. Structuring the Concourse. A pool of statements concerning the issue under investigation is generated.
   2. Structuring the Q set. A formal procedure is used to choose a smaller number of statements to which participants will be asked to respond.
   3. Selection of participants (P-set)
2. Data Collection: Participants are asked to sort the statements to a scale of ‘Mostly Agree’ to ‘Mostly Disagree’ on a template (the ‘grid’) designed to force responses to the form of a quasi normal distribution in order to facilitate comparison between individual Q sorts.
3. Data Analysis. Data are analyzed (using Principal Component Analysis) from several statement sortings to discern discourses among respondents.
4. Results. Factors (discourses) are identified and associated to statements; general characteristics of the chosen solution are presented (e.g. % of variability explained, etc.).
5. Interpretation. Results are verbally interpreted to outline discourse characteristics by specifying issues that each discourse mostly supports and rejects, by highlighting issues that differentiate each discourse from the rest, and issues of consensus among discourses.
6. **Study Design**
   1. **Concourse**

In Q-studies, the first step is to build the ‘concourse’, i.e. obtain the full range of views on a given topic^2^. In this study, the concourse consisted of justifications for eating meat in light of environmental and animal welfare impacts. The concourse was compiled using justifications in the media, popular texts (e.g.: non-fiction on the subject of meat-consumption or vegetarianism), academic literature ^18–20^, as well as informal preliminary interviews with three postgraduate students and one professor in environmental sciences.

Next, the concourse was parsed into single written statements, ensuring that those were short, clear, and represented a single view about the topic of investigation. Conversion to statements was achieved by compiling a list of all the justifications found in the concourse, eliminating identical statements, and shortening lengthier ones. This yielded a list of 116 statements for the study’s concourse, or else the study’s ‘opinion domain’^21^ in the sense of the range of opinions available about meat eating. To establish this domain at 116 statements, we employed a saturation principle to decide when to stop generating more statements.

- 1. **Q-set**

To administer Q, the concourse needs to be reduced to a manageable sample, which should be ‘broadly representative’ of the full range of opinions on the topic^21^. To produce our Q-set, we used formal procedures. Specifically, and following previous studies^9^, we employed a two-axed ‘concourse matrix’, a heuristic device which was used to place statements along a continuum of categories that captured in an exhaustive way the diversity of available opinions on the topic of meat-eating.

The x-axis of our concourse matrix (Figure 1) used the 4Ns of meat-eating justification identified in Piazza et al^18^ (natural, necessary, normal, nice). However, these categories were insufficient to capture the full range of justifications we found in the process of concourse generation, and through a process of open coding, additional categories were created to encompass the remaining statements. Those categories were ‘ethico-philosophical’, ‘systemic’, and ‘environmental’, reflecting the main emphasis, focus or normative target of the statements.

The y-axis of the concourse matrix (Figure 1) tried to capture the plurality of potential rationales that could lie behind consumption choices. To achieve this, we used categories of rational action as those are defined in the expanded model of human rationality developed by Hargreaves-Heap^22^.This framework, which has been used in other Q-studies^23^ includes: instrumental rationality, which understands action as the most effective means to satisfy a given set of ends; procedural rationality, where actions adhere to particular procedures, social norms, or rules of thumb; and, expressive rationality, which understands action as conveying meaning and indicating what people value in life, a set of judgements about what is worthy.

**Figure 1: The concourse matrix**

| *Rationality/4Ns* | Natural | Necessary | Normal | Nice | Ethico-Philosophical | Systemic | Environmental |
| --- | --- | --- | --- | --- | --- | --- | --- |
| Instrumental |  |  |  |  |  |  |  |
| Procedural |  |  |  |  |  |  |  |
| Expressive |  |  |  |  |  |  |  |

Final statements were edited for simplicity and clarity, and to represent diversity of viewpoints and the initial proportion of statements in each position of the concourse matrix. Two pilot Q-sorts were conducted and ambiguous or otherwise problematic statements were edited, reworded, or replaced, while preserving the same number of statements in each category. Thirty statements were chosen as an appropriate final number of statements, given the relatively high planned number of interviews (40), and the exponentially increasing complexity and time required for participants to sort more statements. Both the number of statements and participants are within standard ranges in Q-methodology studies^2,24^.

**Table 1: Q Statements**

| No. | Statement |
| --- | --- |
| 1 | Animals don't suffer as much as we do because they are less complex organisms. |
| 2 | It would be too expensive for me to eat well and be vegetarian. |
| 3 | Animals raised for meat wouldn't have a life at all if it weren't for us. |
| 4 | A lot of people would lose their jobs if we all stopped eating meat. |
| 5 | The environmental impacts of eating meat are exaggerated. |
| 6 | We would all have to stop together for it to make a difference. |
| 7 | I focus my energy on solving more important problems than the environmental impacts of meat. |
| 8 | Widespread vegetarianism would have a negative impact on the environment. (For example, hunters support conservation far more than animal rights activists.) |
| 9 | Other animals eat each other and it’s natural. Humans are also part of the life-cycle. |
| 10 | It's too difficult to prepare meals and get all the right nutrients without meat. |
| 11 | I grew up eating meat and it’s part of my culture. |
| 12 | There are humane methods of raising and slaughtering animals. |
| 13 | Animals are not capable of morality, so we don’t need to give them moral status. |
| 14 | There are sustainable alternatives to industrial farming. |
| 15 | We have evolved to eat meat and have the biology of omnivores (meat-eating teeth, digestive tracts, etc.) |
| 16 | Enjoying meat with others is important to me. |
| 17 | Plants have as much right to exist and be free from harm as animals do. |
| 18 | Changing industrial farming is a matter of political change, not individual choice. |
| 19 | Technology will solve the environmental and animal welfare problems of meat production (ex: in vitro meat). |
| 20 | It would be socially awkward for me to stop eating meat. |
| 21 | It's natural because we're high on the food chain. |
| 22 | I realize I have to stop in the future. |
| 23 | I don't want to be identified as a vegan/vegetarian because they're not likeable. |
| 24 | Because I’m distant from food animals it´s hard to connect my consumption and their suffering. |
| 25 | I feel like it’s wrong to eat meat, but I’m not so good a person that I can give it up entirely. |
| 26 | Meat is convenient and readily available. |
| 27 | It’s not my fault the planet is so crowded we have resorted to industrial farming. Why should I have to give up eating meat? |
| 28 | Vegetarianism/veganism reinforces the illusion that we can be separate from nature. |
| 29 | Eating meat shows that we are affluent in our society. |
| 30 | Meat is delicious. |

# **Participant Selection (P-set)**

Through a combination of purposive and snowball sampling, 42 participants were selected from the Autonomous University of Barcelona’s environmental research center, the Institute of Environmental Science and Technology (ICTA-UAB). These constituted our study’s participant or person set (P-set).

Purposive sampling is a type of non-probability sampling used when sampling with a purpose in mind, a purpose related to the nature, focus, theoretical motivations, or target of the study. For example, market research studies may use purposive sampling when interested in eliciting the views, practices, activities, etc. of a specific sub-group of the population with the aim of marketing specific products to them.

Our P-set selection strategy follows established practice and theory in Q methodology^21^. Q typically selects participants based on diversity of perspectives and knowledge on the topic under investigation rather than on quantity or representativeness of backgrounds^2^. ICTA’s researchers fit the research aim’s profile of interrogating the subjective viewpoints of highly knowledgeable and environmentally aware meat-eaters.

To further pursue diversity, individual participants were also selected on the basis of diversity in terms of gender, nationality, age group, disciplinary background, and level of education, with the expectation that more diverse discourses would be represented as a result. Participants were aged 23-64, from Masters students developing their research thesis project to senior researchers. Participants stated their frequency of meat consumption from once per week to more than once per day, eating organic, high-welfare, as well as industrially produced meat.

**Figure 2: The P-set**

|  | **Number** | **%** |
| --- | --- | --- |
| **Professional Status** |  |  |
| Interns | 2 | 4.8 |
| Masters Students | 5 | 11.9 |
| PhD Candidates | 16 | 38.1 |
| Post-Doctoral Candidates | 9 | 21.4 |
| Professors | 10 | 23.8 |
|  |  |  |
| **Times meat consumed/week** | 3.5 | 50.0 |
|  |  |  |
| **Age Group** |  |  |
| 20-29 | 19 | 45.2 |
| 30-39 | 12 | 28.6 |
| 40-49 | 3 | 7.1 |
| 50-59 | 4 | 9.5 |
| 60-65 | 4 | 9.5 |
|  |  |  |
| **Average Age** | 36 |  |
|  |  |  |
| **Gender** |  |  |
| Female | 18 | 43 |
| Male | 24 | 57 |
|  |  |  |
| **Country of Origin** |  |  |
| Spain | 24 | 57.1% |
| U.S. | 4 | 9.5% |
| Brazil | 2 | 4.8% |
| Canada | 2 | 4.8% |
| Italy | 2 | 4.8% |
| Mexico | 2 | 4.8% |
| France | 1 | 2.4% |
| Germany | 1 | 2.4% |
| Greece | 1 | 2.4% |
| India | 1 | 2.4% |
| Italy/Scotland | 1 | 2.4% |
| Peru | 1 | 2.4% |

# **Data Collection**

Forty-two individual face-to-face Q-interviews were conducted in-situ from May to June 2017. The project was briefly introduced, and an information and consent form was given to participants. Participants had a chance to ask questions about the study before signing. Respondents were then given statement cards in a random order and asked to assign the statements a value, placing them on the grid based on level of agreement/disagreement (“mostly agree” to “mostly disagree”). Caution was taken to explain that participants should sort the cards based on their personal reasons for eating meat, and try to give an honest representation of their own justifications rather than judging the statements solely based on perceived truth or falsity. Participants were not given a fixed amount of time to complete the task. When they finished placing the cards, they were asked to check if the sort accurately represented their viewpoint, and encouraged to move any statements accordingly. Following this, a post-sorting interview was conducted. This consisted of asking participants why they made certain choices, primarily focusing on the highest (+3/+4) and lowest (-3/-4) ranked statements, or unexpected placements. Before concluding, participants were asked if they wanted to comment on any other statements in particular; if they would add any statements to the Q-set; and for general feedback on their experience. Interviews lasted between 40 minutes to 1.5 hours, typically 1 hour.

The shape of the Q-grid (Figure 3) for this study mimicked a standard distribution, as is conventional in most Q-studies^2^. Scores ranged from -4 to +4.

**Figure 3: The Grid**

|  | Most unlike my view |  |  |  | Neutral / Not Sure |  |  |  | Most like my view |  |
| --- | --- | --- | --- | --- | --- | --- | --- | --- | --- | --- |
|  | -4 | -3 | -2 | -1 | 0 | +1 | +2 | +3 | +4 |  |
|  |  |  |  |  |  |  |  |  |  |  |
|  |  |  |  |  |  |  |  |  |  |  |
|  |  |  |  |  |  |  |  |  |  |  |
|  |  |  |  |  |  |  |  |  |  |  |
|  |  |  |  |  |  |  |  |  |  |  |
|  |  |  |  |  |  |  |  |  |  |  |
|  |  |  |  |  |  |  |  |  |  |  |

# **Data Analysis**

Using the statistical technique of factor analysis which reduces data variability by identifying latent variables in the data, Q-methodology extracts factors based on similarities between individual sorts, and generates scores and other data that represent each factor^3^. Factors are then taken to represent distinct discourses. For example, in our study, each factor incorporates a distinct pattern of justifications for eating meat.

We used the freely available software PQMethod to conduct our analysis. Our study applied Principal Component Analysis (PCA) and Varimax rotation, currently the most common techniques used in Q^25^. Our data analysis produced a 4-factor solution. In what follows, and following rules set for good practice in reporting Q studies^24^, we explain the criteria and processes we used to arrive at that solution.

Many Q proponents advocate centroid/manual rotation on the basis that it is most appropriate for allowing the researcher to decide upon the most *meaningful* solution, a core tenet of Q methodology^1,2,25^. For this reason, we initially opted to compute our factors combining PCA and manual rotation. This strikes a balance between finding solutions based on meaningfulness and finding the most statistically desirable solution^1^. However, manual rotations were equally or less meaningful, while yielding similar, but ultimately inferior statistical results. This is a common result of manual rotation^2^.

The 4-factor solution was decided upon after rotating and examining 2-7 factor solutions using both PCA/Varimax rotation and PCA/Manual rotation. Those other solutions produced unloaded factors (factors with no sorts correlating significantly with them), and higher numbers of confounded or unloaded sorts (sorts that did not load significantly on *any* factor). A major concern was that other solutions produced very high inter-factor correlations, which generally indicates a level of similarity between factors that makes them difficult to interpret and less meaningful^3^.

Loadings were considered significant at (p < .01) if they exceeded ±.472 (see Table 2), following a formula typically used in Q-studies, 2.58(1/√n) (where n = the total number of statements)^2^. Significant loadings were flagged provided they only loaded significantly on one factor. The 2.58(1/√n) formula was also used to establish a threshold for acceptable inter-factor correlation, as is conventional^2^. Since some inter-factor correlations were initially above this threshold, the threshold for significance was raised slightly (±.52), a procedure recommended in McKeown and Thomas^2^. While this significantly improved correlations, in one case, inter-factor correlation (between Factors 3 and 4) still exceeds this threshold (Table 3). However, degrees of inter-factor correlation here are still consistent with or lower to those of most published Q-methodology studies.

# **Results**

The 4-factor solution explains 58% of the total variance.

Table 2 presents factor loadings. Eight sorts loaded significantly for each of Factor 1 and Factor 2, and 13 sorts for each of Factor 3 and Factor 4. Four sorts were flagged for each of Factor 1 and Factor 2, and eight sorts for each of Factor 3 and Factor 4.

Factor 1 includes five distinguishing statements (three at significance p < .01, two at significance p < .05); Factor 2 includes seven distinguishing statements (two at p < .01, five at p < .05); Factor 3 includes seven distinguishing statements (five at p < .01, two at p < .05); and, Factor 4 incudes six distinguishing statements (two at p < .01, four at p < .05).

There was one consensus statement (Table 6), specifically statement number 7 “I focus my energy on solving more important problems than the environmental impacts of meat”, which was scored between -1 and +1 by the factors.

# **Interpretation**

Factor arrays (Table 4), along with each factor’s distinguishing statements, provided the raw material used to interpret factors, where each factor represented a distinctive discourse. Post-sort interview data was used to confirm or elaborate these factor interpretations.

In this article, factor interpretations are presented in a condensed form (to comply with journal word-count requirements) and in the Main Text of this article. In this Supplementary Information section, we provide support material related to our findings and interpretations by means of factor arrays with normalised scores for each factor (Table 4); salient statements, which indicate both the differences between discourses (i.e. the distinguishing statements for each discourse) and those statements marked at the extremes (of agreement and disagreement) by each discourse (Table 5); and consensus statements, which indicate commonalities between discourses (Table 6). That material, generated during the data analysis stage, has been the basis for conducting our interpretation of factors. Additionally, we provide a sample of qualitative evidence from post-Q-sorting interviews (quotes), which was used as complementary data for interpreting discourses (Table 7).

In the Main Text of the article, we have been selective in the elements (statements) we use to present the four discourses. Specifically, that presentation focuses primarily on distinguishing statements in order to provide a sharper description and explanation of each factor. Nevertheless, during the analysis stage, our interpretation of each discourse has been based on examining all salient statements (Table 5) as is common practice in Q-studies.

**Table 2: Factor Loading Matrix.** Significantly loading sorts indicated by bold font. Flagged sorts indicated with an X. Flagged sorts are those included in the final calculation of the factor array.

| **Factor Loading Matrix** | | | | |
| --- | --- | --- | --- | --- |
|  | Factor 1 | Factor 2 | Factor 3 | Factor 4 |
| Q-Sort Number |  |  |  |  |
| 1 | 0.3143 | 0.1019 | **0.6923X** | 0.2110 |
| 2 | 0.0011 | 0.2564 | **0.7108X** | -0.0076 |
| 3 | 0.3708 | **0.6048X** | 0.1105 | 0.2665 |
| 4 | 0.1799 | 0.3634 | 0.1793 | 0.4360 |
| 5 | 0.2228 | 0.3893 | **0.6145X** | 0.2736 |
| 6 | **0.5805** | **0.5174** | 0.1606 | -0.0672 |
| 7 | 0.1823 | 0.2932 | 0.0385 | **0.6644X** |
| 8 | 0.3757 | 0.3939 | 0.3324 | 0.4091 |
| 9 | 0.1249 | 0.0382 | 0.3986 | 0.3727 |
| 10 | -0.1085 | 0.4526 | **0.4725** | **0.5024** |
| 11 | 0.2924 | 0.3338 | **0.7462X** | 0.0936 |
| 12 | 0.2357 | 0.2096 | 0.4047 | **0.6734X** |
| 13 | 0.3363 | **0.4775** | **0.5956** | 0.2689 |
| 14 | **0.5549** | -0.0004 | **0.4889** | 0.0018 |
| 15 | 0.2991 | 0.2786 | 0.0989 | **0.4921** |
| 16 | **0.5260** | 0.2331 | 0.2665 | 0.3505 |
| 17 | -0.0841 | -0.1275 | 0.3903 | **0.7578X** |
| 18 | **0.5161** | 0.0184 | 0.4449 | **0.5602** |
| 19 | 0.2966 | 0.1641 | 0.2189 | **0.5224X** |
| 20 | 0.0277 | 0.0826 | **0.6469** | **0.5680** |
| 21 | 0.1050 | -0.0482 | **0.6648X** | 0.1960 |
| 22 | **0.6904X** | 0.0195 | 0.2091 | 0.1596 |
| 23 | 0.1905 | **0.4784** | 0.2020 | 0.3468 |
| 24 | **0.5094** | 0.0837 | 0.1515 | **0.4772** |
| 25 | 0.1456 | 0.0997 | 0.2229 | **0.7222X** |
| 26 | 0.0755 | **0.6016X** | 0.3199 | 0.3520 |
| 27 | -0.1471 | 0.4680 | 0.4501 | 0.3641 |
| 28 | -0.3589 | **0.6911X** | 0.3828 | -0.1345 |
| 29 | 0.2286 | 0.3470 | 0.4459 | 0.1616 |
| 30 | 0.0022 | 0.2451 | **0.555** | **0.6499** |
| 31 | 0.1584 | **0.5853X** | -0.1453 | 0.1991 |
| 32 | **0.5673X** | 0.2927 | 0.3283 | -0.1606 |
| 33 | **0.5639X** | 0.2344 | 0.0071 | 0.3528 |
| 34 | 0.0550 | 0.4378 | 0.1309 | **0.5736X** |
| 35 | 0.4065 | **0.5091** | 0.0230 | 0.2623 |
| 36 | 0.3529 | 0.3069 | 0.0028 | **0.7150X** |
| 37 | -0.0863 | 0.1676 | **0.6370X** | 0.3656 |
| 38 | 0.0691 | -0.1077 | **0.7119X** | 0.2745 |
| 39 | **0.6408X** | -0.0229 | -0.1699 | 0.3031 |
| 40 | 0.2477 | 0.1755 | 0.2864 | **0.7659X** |
| 41 | 0.2263 | 0.3210 | 0.3302 | 0.454 |
| 42 | 0.4034 | 0.1196 | **0.5874X** | 0.2434 |
| Significantly Loading | 8 | 8 | 13 | 13 |
| Flagged | 4 | 4 | 8 | 8 |
| % of explained variance | 11 | 11 | 17 | 18 |
| Total Unloaded Sorts | 7 | | | |
| Total Confounded Sorts | 7 | | | |

**Table 3: Factor Correlation Matrix.**

| **Factor Correlation Matrix** | | | | |
| --- | --- | --- | --- | --- |
| Factor | 1 | 2 | 3 | 4 |
| 1 | 1 | 0.2374 | 0.4226 | 0.4654 |
|  |  |  |  |  |
| 2 |  | 1 | 0.4631 | 0.4371 |
|  |  |  |  |  |
| 3 |  |  | 1 | 0.5791 |
|  |  |  |  |  |
| 4 |  |  |  | 1 |

**Table 4: Q-statements and Factor Array.** Distinguishing statements marked by one asterisk (*) for significance at p < .01 or two asterisks (**) for significance at p < .05.

| **No.** | **Statement** | **Factor 1** | | **Factor 2** | | **Factor 3** | | **Factor 4** | |
| --- | --- | --- | --- | --- | --- | --- | --- | --- | --- |
|  |  | *score* | *z-score* | *score* | *z-score* | *score* | *z-score* | *score* | *z-score* |
| 1 | Animals don't suffer as much as we do because they are less complex organisms. | -2 | -1.24 | 0* | -0.03 | -4* | -2.37 | -2 | -0.95 |
| 2 | It would be too expensive for me to eat well and be vegetarian. | -2 | -0.65 | -3 | -1.37 | 0** | 0 | -1 | -0.89 |
| 3 | Animals raised for meat wouldn't have a life at all if it weren't for us. | 0 | -0.03 | -1 | -0.38 | -2 | -0.97 | -2 | -0.91 |
| 4 | A lot of people would lose their jobs if we all stopped eating meat. | -2 | -1.26 | -1 | -0.44 | 1* | 0.4 | -3 | -1.23 |
| 5 | The environmental impacts of eating meat are exaggerated. | -3 | -1.52 | 3* | 1.5 | -1* | -0.62 | -3 | -1.46 |
| 6 | We would all have to stop together for it to make a difference. | 2 | 0.76 | 1 | 0.36 | -1 | -0.58 | -1 | -0.41 |
| 7 | I focus my energy on solving more important problems than the environmental impacts of meat. | 1 | 0.19 | -1 | -0.32 | 0 | 0.07 | 0 | -0.16 |
| 8 | Widespread vegetarianism would have a negative impact on the environment. (For example, hunters support conservation far more than animal rights activists.) | 0 | 0.14 | 0 | 0.17 | 0 | 0.05 | -1** | -0.89 |
| 9 | Other animals eat each other and it’s natural. Humans are also part of the life-cycle. | 0* | -0.21 | 1 | 0.81 | 2 | 1.11 | 2 | 1.13 |
| 10 | It's too difficult to prepare meals and get all the right nutrients without meat. | -1 | -0.38 | 2 | 0.95 | 0 | -0.2 | 1 | 0.33 |
| 11 | I grew up eating meat and it’s part of my culture. | 2 | 1.04 | 1 | 0.46 | 3 | 1.83 | 3 | 1.57 |
| 12 | There are humane methods of raising and slaughtering animals. | 2 | 0.77 | 2 | 1.11 | 2 | 0.98 | 0 | 0.23 |
| 13 | Animals are not capable of morality, so we don’t need to give them moral status. | -3 | -1.27 | -1 | -0.76 | -2 | -0.96 | -1 | -0.65 |
| 14 | There are sustainable alternatives to industrial farming. | 4 | 2.52 | 4 | 2.07 | 4 | 1.98 | 1** | 0.85 |
| 15 | We have evolved to eat meat and have the biology of omnivores (meat-eating teeth, digestive tracts, etc.) | -1** | -0.5 | 0** | 0.23 | 3 | 1.42 | 3 | 1.24 |
| 16 | Enjoying meat with others is important to me. | 0 | -0.17 | 3** | 1.38 | 1 | 0.23 | 1 | 0.78 |
| 17 | Plants have as much right to exist and be free from harm as animals do. | 1 | 0.68 | -1 | -0.83 | 1 | 0.33 | -1 | -0.51 |
| 18 | Changing industrial farming is a matter of political change, not individual choice. | 0 | 0.18 | 2** | 1.13 | -1** | -0.45 | 1 | 0.44 |
| 19 | Technology will solve the environmental and animal welfare problems of meat production (ex: in vitro meat). | 3* | 1.41 | -4** | -1.79 | 0* | -0.38 | -2** | -1.07 |
| 20 | It would be socially awkward for me to stop eating meat. | 1 | 0.35 | 1 | 0.26 | -3* | -1.41 | 0 | 0.09 |
| 21 | It's natural because we're high on the food chain. | -2 | -0.95 | 0 | -0.28 | 1 | 0.67 | 0 | 0.25 |
| 22 | I realize I have to stop in the future. | 3* | 1.53 | 0 | -0.02 | 0 | -0.11 | 1 | 0.39 |
| 23 | I don't want to be identified as a vegan/vegetarian because they're not likeable. | -1 | -0.54 | -2 | -0.99 | -1 | -0.41 | -4* | -1.86 |
| 24 | Because I’m distant from food animals it´s hard to connect my consumption and their suffering. | 0 | -0.17 | -2 | -0.95 | -2 | -0.75 | 2** | 1.15 |
| 25 | I feel like it’s wrong to eat meat, but I’m not so good a person that I can give it up entirely. | 2 | 1.17 | -2 | -0.98 | -1 | -0.62 | 2 | 1.01 |
| 26 | Meat is convenient and readily available. | 1 | 0.61 | 0 | 0.23 | 2 | 0.95 | 2 | 1.22 |
| 27 | It’s not my fault the planet is so crowded we have resorted to industrial farming. Why should I have to give up eating meat? | -4 | -1.94 | -2 | -1.34 | -3 | -1.05 | -2 | -1.13 |
| 28 | Vegetarianism/veganism reinforces the illusion that we can be separate from nature. | -1 | -0.54 | 1 | 0.39 | 1 | 0.57 | 0 | -0.24 |
| 29 | Eating meat shows that we are affluent in our society. | -1 | -0.45 | -3** | -1.72 | -2 | -1.02 | 0 | -0.34 |
| 30 | Meat is delicious. | 1** | 0.46 | 2 | 1.16 | 2 | 1.31 | 4* | 2.02 |

***Table 5: Four Discourses and their Salient Statements.*** *Salient statements include the strongest agree/disagree statements (+3, +4, -3, -4) and distinguishing statements, in light grey marked by one asterisk (*) (Significance at p < .01), or deep grey marked by two asterisks (**) (Significance at p < .05).*

| **No.** | **Salient Statements for Factor** | **Score** | **Z-score** |
| --- | --- | --- | --- |

***Discourse 1 (D1). Optimism: “The future will solve it.”***

| 14 | There are sustainable alternatives to industrial farming. | +4 | 2.520 |
| --- | --- | --- | --- |
| 22* | I realize I have to stop in the future. | +3 | 1.533 |
| 19* | Technology will solve the environmental and animal welfare problems of meat production (ex: in vitro meat). | +3 | 1.414 |
| 11 | I grew up eating meat and it’s part of my culture. | +2 | 1.044 |
| 30** | Meat is delicious. | +1 | 0.464 |
| 9* | Other animals eat each other and it’s natural. Humans are also part of the life-cycle. | 0 | -0.209 |
| 15** | We have evolved to eat meat and have the biology of omnivores | -1 | -0.501 |
| 13 | Animals are not capable of morality, so we don’t need to give them moral status. | -3 | -1.270 |
| 5 | The environmental impacts of eating meat are exaggerated. | -3 | -1.523 |
| 27 | It’s not my fault the planet is so crowded we have resorted to industrial farming. Why should I have to give up eating meat? | -4 | -1.936 |

***Discourse 2 (D2). System Focus: “It’s the system, not the meat!”***

| 14 | There are sustainable alternatives to industrial farming. | +4 | 2.066 |
| --- | --- | --- | --- |
| 5* | The environmental impacts of eating meat are exaggerated. | +3 | 1.500 |
| 16** | Enjoying meat with others is important to me. | +3 | 1.381 |
| 18** | Changing industrial farming is a matter of political change, not individual choice. | +2 | 1.126 |
| 15** | We have evolved to eat meat and have the biology of omnivores. | 0 | 0.235 |
| 1* | Animals don't suffer as much as we do because they are less complex organisms | 0 | -0.030 |
| 2 | It would be too expensive for me to eat well and be vegetarian. | -3 | -1.365 |
| 29** | Eating meat shows that we are affluent in our society. | -3 | -1.723 |
| 19** | Technology will solve the environmental and animal welfare problems of meat production (ex: in vitro meat). | -4 | -1.791 |

***Discourse 3 (D3). Complexity: “It is my moral responsibility, but it’s more complex than that…”***

| 14 | There are sustainable alternatives to industrial farming. | +4 | 1.984 |
| --- | --- | --- | --- |
| 11 | I grew up eating meat and it’s part of my culture. | +3 | 1.830 |
| 15 | We have evolved to eat meat and have the biology of omnivores. | +3 | 1.418 |
| 4* | A lot of people would lose their jobs if we all stopped eating meat. | +1 | 0.398 |
| 2** | It would be too expensive for me to eat well and be vegetarian | 0 | -0.000 |
| 19* | Technology will solve the environmental and animal welfare problems of meat production (ex: in vitro meat). | 0 | -0.382 |
| 18** | Changing industrial farming of meat is a matter of political change, not individual choice. | -1 | -0.453 |
| 5* | The environmental impacts of eating meat are exaggerated. | -1 | -0.616 |
| 27 | It’s not my fault the planet is so crowded we have resorted to industrial farming. Why should I have to give up eating meat? | -3 | -1.048 |
| 20* | It would be socially awkward for me to stop eating meat. | -3 | -1.408 |
| 1* | Animals don't suffer as much as we do because they are less complex organisms | -4 | -2.365 |

***Discourse 4 (D4). Feebleness: “I should, but I lack the willpower”***

| 30* | Meat is delicious. | +4 | 2.025 |
| --- | --- | --- | --- |
| 11 | I grew up eating meat and it’s part of my culture. | +3 | 1.571 |
| 15 | We have evolved to eat meat and have the biology of omnivores | +3 | 1.242 |
| 24** | Because I’m distant from food animals it´s hard to connect my consumption and their suffering. | +2 | 1.146 |
| 14** | There are sustainable alternatives to industrial farming. | +1 | 0.846 |
| 8** | Widespread vegetarianism would have a negative impact on the environment. (For example, hunters support conservation far more than animal rights activists.) | -2 | -0.889 |
| 19** | Technology will solve the environmental and animal welfare problems of meat production (ex: in vitro meat). | -2 | -1.071 |
| 4 | A lot of people would lose their jobs if we stopped eating meat. | -3 | -1.228 |
| 5 | The environmental impacts of eating meat are exaggerated. | -3 | -1.464 |
| 23* | I don’t want to be identified as a vegan/vegetarian because they’re not likeable. | -4 | -1.856 |

**Table 6: Consensus Statements.** Consensus statements indicated with one asterisk (*) at p < .01 and two asterisks (**) p < .05

| **No.** | **Statement** | **Score** |
| --- | --- | --- |
| 7* | I focus my energy on solving more important problems than the environmental impacts of meat. | -1 to +1 |

**Table 7: Salient quotes from interviews in each group**

| **Factor 1** | |
| --- | --- |
| **Interview** | **Quote** |
| #I22 | “No it’s not [natural because we’re higher on the food chain], because we are part of that environment, and it’s not a pyramid. That’s why the food chain works, because we are all connected, and we are all part of one space.” |
| #I32 | “I think that we have to start to change the upper part of the pyramid. If we can change the upper part then [it would] change the lower levels.”  “[industrially farmed animals] grow up in a few weeks… we have the same right to be alive… […] in my opinion we are on the same level.” |
| #I33 | “[I believe in] Collective change… individual change, like this drop to drop change is so slow, so I would say community change. Like for example this block of buildings that decided, ‘ok you know what, let’s put the common fridge for the building… so we save energy or whatever. Or let’s put solar panels on our roof…” |
| **Factor 2** | |
| **Interview** | **Quote** |
| #I26 | “Technology is not gonna be the solution for the industrial farming, particularly because the industrial farming sector doesn’t care about the way animals are growing, so they are not going to demand better technology for animals growing in better conditions, so it’s not a topic that really worries.. politicians or the powerful people in the agro-industrial sector and the meat sector… so technology is not going to be focused on improving the conditions of animals. They will probably create technology in order to make the sector more efficient in terms of economic gains and so on… ” |
| #I28 | “The goal is to change completely… I think it’s a political issue and an issue of capitalism. And actually I think it’s worse [to not eat meat] because you convince yourself that you’re doing something good. So you’re like ‘oh, I’m not eating meat, I’m so environmentally friendly… and you don’t focus on other issues which are at the core of everything and the consumption side is actually not going to change anything.” |
| **Factor 3** | |
| **Interview** | **Quote** |
| #I2 | “When you get to a really big scale, it’s very difficult to establish a relationship of causality between what you do and what happens. There are too many variables […] It’s nice to imagine that we are being nice and saving the world but it’s complicated, especially with food…”  “It doesn’t matter if it makes a difference, no. You, you have to take responsibility for yourself.” |
| #I11 | “Every choice has an impact, everything has an effect…” |
| #I37 | “Consumers should take responsibility for their choices”  “I mean, being responsible in my view is more complex… trying to actively participate in society against that industrial system… it’s not a yes or a no. It’s how much, when, for what purpose, because it’s not the same. [later] I do eat meat and I’m more responsible than [vegetarians]. [Vegetarians] pretend to save the world… ahh [frustrated] I *do* eat meat. Full stop.” |
| **Factor 4** | |
| **Interview** | **Quote** |
| #I19 | “Unfortunately it’s delicious.” |
| #I25 | “Putting your ideals [above] your own pleasure… making your life difficult for your ideals is something that I admire.” |
| #I34 | “I know I am eating more than I need, because it’s a pleasure […] eating is a part of my culture. That means […] we can sit, we can eat, we can have a barbecue starting from the noon until the night eating, drinking. It’s a pleasure.” |
| #I36 | “I like the taste of meat […] I’ve weighed the alternatives many times.”  “I think they’ve made a less selfish decision than I have, actually.” |

# **References**

1. Watts, S. & Stenner, P. *Doing Q methodological research: Theory, method & interpretation*. (Sage, 2012).

2. McKeown, B. & Thomas, D. *Q Methodology*. (SAGE Publications, Inc., 2013). doi:10.4135/9781483384412

3. Brown, S. R. *Political subjectivity: applications of Q methodology in political science*. (Yale University Press, 1980).

4. Stephenson, W. *The study of behavior: Q-technique and its methodology*. (University of Chicago Press, 1953).

5. Pike, K., Wright, P., Wink, B. & Fletcher, S. The assessment of cultural ecosystem services in the marine environment using Q methodology. *J. Coast. Conserv. Dordr.* **19**, 667–675 (2015).

6. Stevenson, H. Contemporary Discourses of Green Political Economy: A Q Method Analysis. *J. Environ. Policy Plan.* 1–21 (2015). doi:10.1080/1523908X.2015.1118681

7. Crivits, M. *et al.* Scenarios based on sustainability discourses: Constructing alternative consumption and consumer perspectives. *Futures* **42**, 1187–1199 (2010).

8. Masso, M. D. & Zografos, C. Constructing food sovereignty in Catalonia: different narratives for transformative action. *Agric. Hum. Values* **32**, 183–198 (2015).

9. Barry, J. & Proops, J. Seeking sustainability discourses with Q methodology. *Ecol. Econ.* **28**, 337–345 (1999).

10. Zabala, A., Sandbrook, C. & Mukherjee, N. When and how to use Q methodology to understand perspectives in conservation research. *Conserv. Biol.* **32**, 1185–1194 (2018).

11. Robbins, P. & Krueger, R. Beyond bias? The promise and limits of Q method in human geography. *Prof. Geogr.* **52**, 636–648 (2000).

12. McKeown, B. F. & Thomas, D. B. *Q methodology (Quantitative applications in the social sciences series, vol. 66)*. (Thousand Oaks, CA: Sage, 1988).

13. Danielson, S. Q method and surveys: Three ways to combine Q and R. *Field Methods* **21**, 219–237 (2009).

14. Chamberlain, E. C., Rutherford, M. B. & Gibeau, M. L. Human perspectives and conservation of grizzly bears in Banff National Park, Canada. *Conserv. Biol.* **26**, 420–431 (2012).

15. Peritore, N. P. & Karina, A. 10. Ecopolitics in the Global South: AQ Method Study of Elites in Seven Nations. *Soc. Discourse Environ. Policy Appl. Q Methodol.* **196**, (2000).

16. Neff, M. W. What research should be done and why? Four competing visions among ecologists. *Front. Ecol. Environ.* **9**, 462–469 (2011).

17. Sandbrook, C. G., Fisher, J. A. & Vira, B. What do conservationists think about markets? *Geoforum* **50**, 232–240 (2013).

18. Piazza, J. *et al.* Rationalizing meat consumption. The 4Ns. *Appetite* **91**, 114–128 (2015).

19. Šedová, I., Slovák, Ľ. & Ježková, I. Coping with unpleasant knowledge: Meat eating among students of environmental studies. *Appetite* **107**, 415–424 (2016).

20. Veilleux, S. Coping With Dissonance: Psychological Mechanisms That Enable Ambivalent Attitudes Toward Animals. *Univ. Maine Digit. Commons Honors Coll.* **Paper 196**, (2014).

21. Watts, S. & Stenner, P. Doing Q Methodology: theory, method and interpretation. *Qual. Res. Psychol.* **2**, 67–91 (2005).

22. Heap, S. H. *Rationality in economics*. (B. Blackwell, 1989).

23. Baker, R. M. Economic rationality and health and lifestyle choices for people with diabetes. *Soc. Sci. Med.* **63**, 2341–2353 (2006).

24. Rogers, R. S. *Social psychology: A critical agenda*. (1995).

25. Ramlo, S. Centroid and Theoretical Rotation: Justification for Their Use in Q Methodology Research. *-West. Educ. Res.* **28**, (2016).
